# SEXUAL MOTIVES, MARITAL QUALITY, AND DEPRESSIVE SYMPTOMS OF MID-LIFE SAME- AND DIFFERENT-SEX COUPLES

**DOI:** 10.1093/geroni/igad104.1712

**Published:** 2023-12-21

**Authors:** Hye Won Chai, Susanna Joo, Jihye Lee, Debra Umberson

**Affiliations:** University of Texas at Austin, Austin, Texas, United States; BK21. Symbiotic Society and Design, Seoul, Republic of Korea; Yonsei University, Seoul, Republic of Korea; University of Texas at Austin, Austin, Texas, United States

## Abstract

Self-determined sexual motives, or self-endorsed reasons for having sex, is a significant determinant of psychological health with notable gendered patterns. However, previous studies mostly focused on young adult different-sex couples and fell short of exploring the mechanisms through which self-determined sexual motives are associated with psychological health. Using dyadic data from the Health and Relationships Project (HARP) on 124 gay, 171 lesbian, and 124 straight midlife married couples, this study examined the mediating role of marital quality in the association between self-determined sexual motives and depressive symptoms. Actor-partner interdependence mediation model (APIMeM) was used for analyses. Results showed significant mediating effects of individual’s own marital quality for all couple types (i.e., gay, lesbian, straight couples), such that individuals with higher levels of self-determined sexual motives reported better marital quality, which in turn was associated with their lower levels of depressive symptoms. For partner effects, same patterns emerged for lesbian and gay couples wherein individuals’ own marital quality mediated the association between their spouse’s sexual motives and their own depressive symptoms. However, there were gendered patterns within straight couples. For women, their own marital quality mediated the association between their partner’s sexual motives and their own depressive symptoms. For men, their spouse’s marital quality mediated the association between their partner’s sexual motives and their own depressive symptoms. These findings suggest marital quality as a useful intervention point to enhance the psychological health of midlife couples in same-sex and different-sex marriages and also highlight the salience of gendered dynamics within straight couples.

